# Lattice Boltzmann Simulation of Coupling Heat Transfer between Solid and Gas Phases of Nanoporous Materials

**DOI:** 10.3390/nano12193424

**Published:** 2022-09-29

**Authors:** Yafen Han, Shuai Li, Haidong Liu, Yucong Li

**Affiliations:** School of Energy and Power Engineering, Northeast Electric Power University, Jilin 132013, China

**Keywords:** heat transfer, nanomaterials, aerogel, temperature distribution, thermal conductivity

## Abstract

In order to deeply study the heat conduction of nanoporous aerogel, a model of gas-solid heat conduction was established based on the microstructure of aerogel. The model was divided into two subdomains with uniform mesh because of the different gas-solid characteristics, and simulation was performed on each domain using the lattice Boltzmann method. The value of temperature on the boundaries of subdomains was determined by interpolation. Finally, the temperature distribution and the thermal conductivity were maintained. It can be concluded that when the gas-phase scale was fixed, the temperature distribution of the solid phase became more uniform when the scale increased; when the solid-phase scale was fixed, the temperature jump on the gas-solid interface decreased with the increase in the gas-phase scale; and the thermal conductivity of gas-solid coupling varied with the scale of the gas phase or solid phase, showing a scale effect in varying degrees.

## 1. Introduction

Aerogels, as a super insulation material, have a unique structure that is an open nanoporous and continuous three-dimensional skeleton. Therefore, they exhibit outstanding properties such as high porosity, high specific surface area and low thermal conductivity [1]. They have been applied in many fields such as aerospace, industrial insulation and military technology [2]. In the thermal transport of aerogel, the heat needs to continuously pass through the porous network composed of gas and solid. The complex microscopic characteristics and the scale effect of gas and solid make it difficult to study the thermal conductivity. With the depletion of natural resources, all countries in the world are committed to the conservation and efficient use of energy. The insulation requirements of aerogel insulation materials are becoming more and more urgent. Accordingly, it is significant to further study the gas-solid coupling thermal conductivity of aerogels in order to optimize and improve their adiabatic performance.

At present, the researches on the gas-solid coupling heat transfer of aerogel mainly focus on theoretical and numerical calculation methods. In terms of theoretical studies, Swimm et al. proposed a calculation model of coupling the thermal conductivity of aerogels and studied the coupling effect of their effective thermal conductivity [3]. Based on the microstructure of aerogels and the model, Zhao J et al. improved the prediction accuracy of the thermal conductivity of aerogels [4]. Given that the above two models are based on the thermal bridge effect, the calculation process is extremely difficult. In addition, Wei et al. used a cubic array composed of small spheres to establish a gas-solid coupling heat conduction model for aerogels [5]. The calculation results showed that the nanostructure and the nanoscale effect of the solid grains were the main reasons for the extremely low thermal conductivity of the materials. In terms of numerical methods, Coquard R. et al. established a model for the overall heat transfer inside aerogels with cellulose [6]. Under the assumption of “uniform approximation”, the heat-transfer law of gas-solid two phases was studied. Zhao J. et al. established a three-dimensional numerical model of silica aerogel based on the random aggregation structure of porous secondary nanoparticles and studied the overall thermal conductivity of aerogel by considering the coupling thermal conductivity [7].

In recent years, the lattice Boltzmann method (LBM) has been widely used in the field of micro-nanoscale heat transfer [8]. It can not only reveal the heat transfer and the intrinsic mechanism of porous insulating materials, but it can also make up for the shortcomings of the macro methods. In addition, the LBM is high efficiency and stable. It has become a powerful tool for the analysis of micro-scale heat transfer. Song proposed a local-effective-viscosity multirelaxation-time lattice Boltzmann pore-network coupling model (LEV-LBM-PNM) to simulate gas transport in nanoporous media [9]. The proposed LEV-LBM-PNM accurately predicts gas-apparent permeability by accounting for gas slip in irregular pore shapes and surface roughness. Qu established a modified lattice LBM to predict effective thermal conductivity of aerogel materials [10]. The modified LBM scheme introduces an additional coefficient to regulate significant differences in inherent thermal conductivity between solid and gas phases. Kan proposed a random internal morphology and structure generation-growth method [11], The model was imported into the lattice Boltzmann algorithm as a fully resolved geometry and used to investigate the effects on heat transfer in nanoporous materials.

In this paper, the gas-solid coupling heat conduction model is established, and the temperature distribution and thermal conductivity of silica-aerogel gas-solid coupling are simulated by LBM based on the region splitting. The thermal transfer of gas-solid coupling heat conduction is analyzed and discussed in depth.

## 2. Physical Model

**Figure 1:**
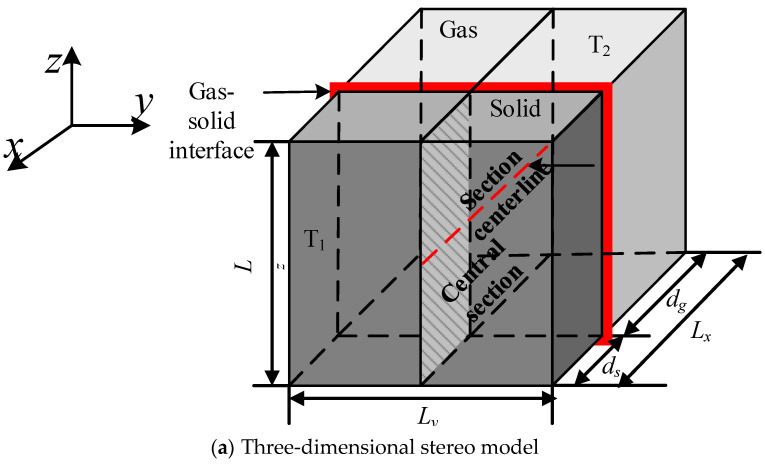

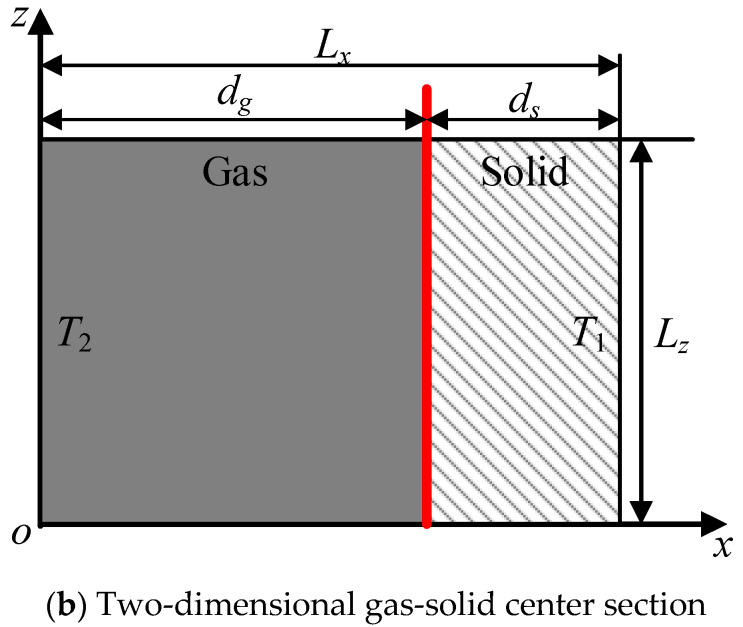
Gas-solid coupled thermal conduction model.

Figure 1 displays the model of gas-solid coupling heat conduction of aerogels. Figure 1a,b are a three-dimensional model and a two-dimensional gas-solid center section, respectively. The model consists of gas-solid two phases, which are air and silica, respectively, and the characteristic scales along the *x* direction are expressed by *d_g_* and *d_s_*. The length, width and height of the model were expressed as *L_x_*, *L_y_*, *L_z_*. The red cross section is the interface of the gas-solid two phases. The front and back walls are kept at a constant temperature; the temperature of the front wall is *T*_1_ = 300 K, and the temperature of the back wall is *T*_2_ = 301 K; the other walls were adiabatic. The heat flow transferred from the high temperature wall (front wall) to the low temperature wall (back wall) along the *x*-axis.

## 3. Mathematical Model

### 3.1. Basic Algorithm

#### 3.1.1. Gas-Phase Basic Algorithm

**Figure 2:**
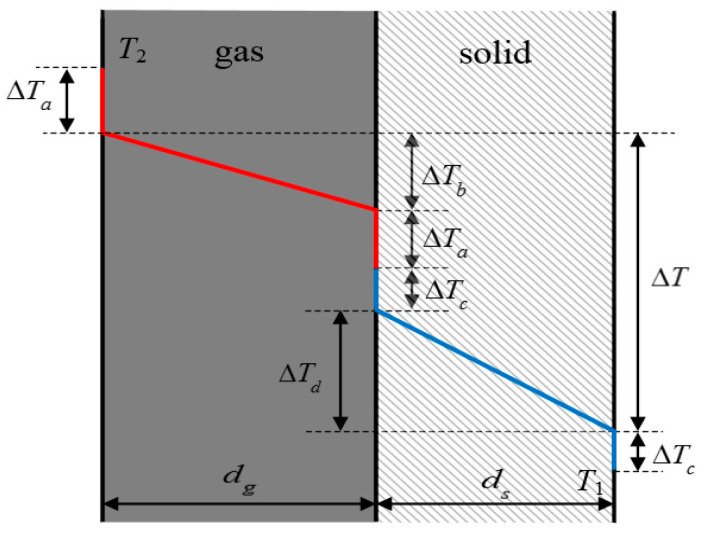
Calculating model of effective thermal conductivity of gas-solid coupling thermal conductivity.

The three-dimensional fifteen-speed (D3Q15) lattice Boltzmann model was used to solve the gas steady heat conduction of aerogel. The evolution equation was as follows:

(1) $$f_{k}(r_{g}+c_{k}\Delta t_{g},t_{g}+\Delta t_{g})-f_{k}(r_{g},t_{g})=-\frac{1}{\tau }[f_{k}(r_{g},t_{g})-f_{k}^{eq}(r_{g},t_{g})]$$

where subscripts *g*, *k* were the gas phase and the discrete velocity directions of the lattice point, respectively; $r_{g}$ was the space position vector, $c_{k}$ was the discrete lattice velocity (as shown in Figure 2); $t_{g}$ was the time, $\Delta t_{g}$ was the time step; τ was the dimensionless relaxation time; $f_{k}$ with $f_{k}^{eq}$ were, respectively, the internal energy distribution function and the corresponding equilibrium distribution function of the discrete velocity directions of the different lattice points in the gas phase.

(2) $$f_{k}^{eq}=\left\{\begin{array}{l} (2/9)\rho c_{p}T\\ (1/9)\rho c_{p}T\\ (1/72)\rho c_{p}T \end{array}\right.\begin{array}{l} k=0\\ k=1∼6\\ k=7∼14 \end{array}$$

where *T* was temperature; *ρ* was the air density; *c_p_* was the specific heat capacity of the air.

(3) $$c_{k}=\left\{\begin{array}{l} (0,0,0)c,k=0\\ (\pm 1,1,0)c,(0,\pm 1,0)c,(0,0,\pm 1)c,k=1∼6\\ (\pm 1,\pm 1,\pm 1)c,k=7∼14 \end{array}\right.$$

(4) $$\tau =\frac{9}{5}\frac{\lambda }{\rho c_{p}c^{2}\delta t}+0.5$$

where *λ* was the thermal conductivity of the air; *c* was the lattice speed that theoretically could take any positive value only to insure the *τ* value within (0.5,2); *c = δx/δt* was the space step [12,13]. Temperature *T* and heat flux *q* could be obtained, respectively, according to the following formula [14]:

(5) $$T=\frac{1}{\rho c_{p}}\sum_{k}f_{k}$$

(6) $$q_{g}=\sum_{k=0}^{Q}\left(c_{k}f\right)\frac{\tau -0.5}{\tau }$$

When the temperature reached equilibrium state, the effective thermal conductivity could be calculated on the temperature *T* and heat flux *q*.

#### 3.1.2. Solid-Phase Basic Algorithm

The three-dimensional fifteen-speed (D3Q15) lattice Boltzmann model was used to solve the solid steady heat conduction of aerogel. The evolution equation was as follow:

(7) $$e_{k}(r_{s}+v_{k}\Delta t_{s},t_{s}+\Delta t_{s})=e_{k}(r_{s},t_{s})[1-\beta ]+\beta e_{k}^{eq}(r_{s},t_{s})$$

where subscripts *s*, *k* were the solid phase and the discrete velocity directions of the lattice point, respectively; $r_{s}$ was the space position vector, $v_{k}$ was the discrete lattice velocity (as shown in Figure 2); $t_{s}$ was the time, $\Delta t_{s}$ was the time step; β was a weight function; $e_{k}$ with $e_{k}^{eq}$ were, respectively, the phonon energy density distribution function and the equilibrium phonon energy density distribution function of the discrete velocity directions of the different lattice points in the solid phase.

The total phonon energy density e is calculated in reference [15]. The total energy density e of each lattice point is discretized into the energy density $e_{k}$ of each discrete direction.

(8) $$e=\sum_{k=1}^{Q}e_{k}$$

Since the phonon energy density e and the equilibrium phonon energy density $e_{k}^{eq}$ of all lattice points need to be redefined at each time step, assuming that the probability of phonon scattering in all directions is equal, i.e. isotropic, the equilibrium phonon energy density

(9) $$e_{k}^{eq}=\frac{1}{Q}\sum_{k=1}^{Q}e$$

(10) $$v_{k}=\left\{\begin{array}{l} (0,0,0)v,k=0\\ (\pm 1,1,0)v,(0,\pm 1,0)v,(0,0,\pm 1)v,k=1\sim 6\\ (\pm 1,\pm 1,\pm 1)v,k=7\sim 14 \end{array}\right.$$

where the lattice speed $v=\Delta r_{s}/\Delta t_{s}$, $\Delta r_{s}$ was the solid-phase space step.

Weight function $\beta =\Delta t_{s}/\tau_{0}$, $\tau_{0}$ was the particle relaxation time.

The normal heat-transfer rate of the *yoz* plane within the model is

(11) $$q_{s}=\sum_{k=0}^{Q}v_{k}\cdot e_{k}(r,t)$$

### 3.2. Boundary Conditions

In the LBM, for calculation accuracy, stability and computational efficiency, the boundary conditions should be reasonably selected according to the actual situation. Since the influences of solid skeleton were not taken into account in the gas heat conduction, the mirror bounce format was adopted for no friction losses. Moreover, the energy distribution function could free transfer in this boundary condition. That was equivalent to removing the boundary, and the results would not be affected by the width $L_{y}$ and the height $L_{z}$. The $L_{x}$ direction is the constant temperature boundaries $T_{1}$ and $T_{2}$. The specific forms were implemented as follow:

(12) $$f_{i^{\prime }}\left(r_{b},t\right)=f_{i}\left(r_{f},t\right)$$

where $r_{b}$ was the boundary lattice point, $r_{f}=r_{b}-e_{i}\delta t$ were internal lattice point; $f_{i^{\prime }}$ was a mirror-symmetric distribution function of $f_{i}$. The $f_{i}$ could be obtained by the inner lattice points that were adjacent the boundary wall. 

### 3.3. Calculation Method of Effective Thermal Conductivity

**Figure 3:**
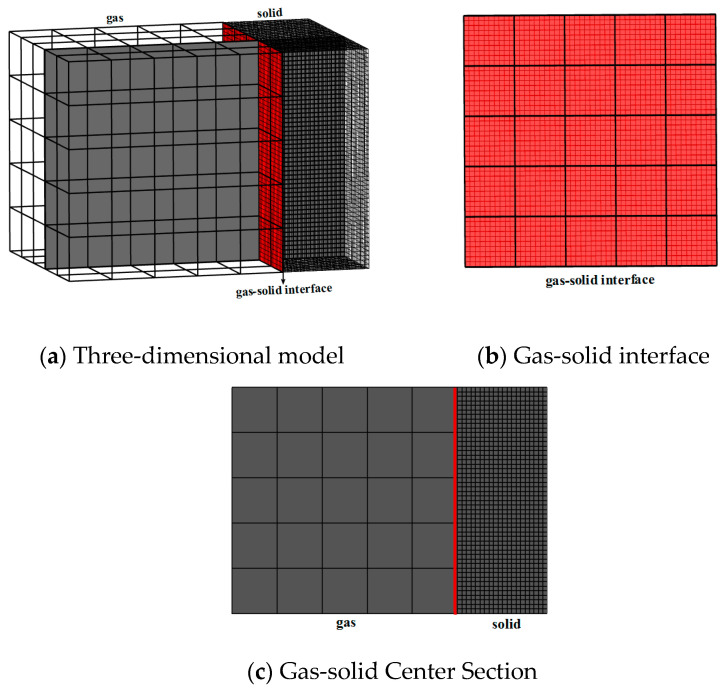
Gas-solid coupled thermal conduction model with lattice partition.

Owing to both the gas-phase region and the solid-phase region being stable and the heat flow in the two sub-regions being equal, *q* is used to represent the heat flow in the whole region, that is *q* = *q*_s_ = *q*_g_. Because the characteristic scales of the whole system region are smaller than or close to the average free path of the gas molecules and phonons, obvious scale effects appear in both sub-regions, and the temperature distribution of materials is no longer continuous. The effective thermal conductivity can be obtained according to Fourier’s law and the geometric relationship in Figure 3.

(13) $$k_{\mathrm{eff}}=\frac{q\cdot (d_{g}+d_{s})}{\Delta T_{a}+\Delta T+\Delta T_{c}}$$

where q is the system heat flow; $\Delta T_{a}$ is the jumping for the gas-phase boundary temperature; $\Delta T_{c}$ is the jumping for the solid-phase boundary temperature.

### 3.4. Region Splitting

Although the hybrid LBM has been proven to provide great advantages over the structured LB in terms of geometry flexibility and the treatment of interfaces [16,17], the focus of this paper is to study the performance of gas-solid coupling heat transfer at the interface. Hence, considering the accuracy and simplicity, the “region splitting” is selected.

The basic idea of region splitting is to divide the whole system region into different sub-regions, and then using the corresponding LBM model to calculate each sub-region independently [18].

**Figure 4:**
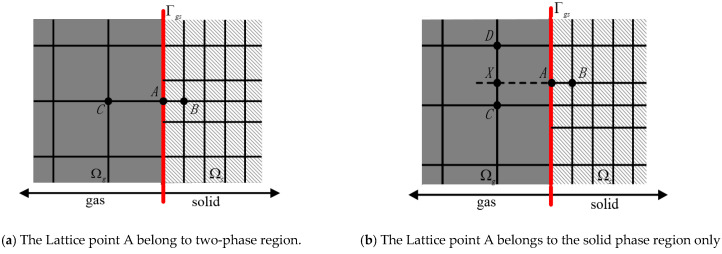
Boundary lattice interpolation of two-dimensional gas-solid center section.

According to the gas-solid coupled thermal conduction model, the gas-solid coupled thermal conduction model is divided into two sub-regions: the gas phase and the solid phase, which are represented by $\Omega_{g}$ and $\Omega_{s}$, $\Gamma_{as}=\Omega_{a}\cap \Omega_{s}$, because the average free path of a gas molecule (about 70 nm) is much larger than that of a phonon (about 0.6 nm) of silica solid. To simulate the heat transfer at the solid boundary, we set a virtual boundary node at the gas region *XA*, as shown in Figure 4. Therefore, different size meshes are used to subdivide the sub-regions, which are set up as $\Delta x_{a}=\Delta y_{a}=\Delta z_{a}$, $\Delta x_{s}=\Delta y_{s}=\Delta z_{s}$. Generally, the time step and space step in the gas-phase and solid-phase regions are different, so the physical quantities involved in the virtual boundary need to be calculated by special methods.

**Figure 5:**
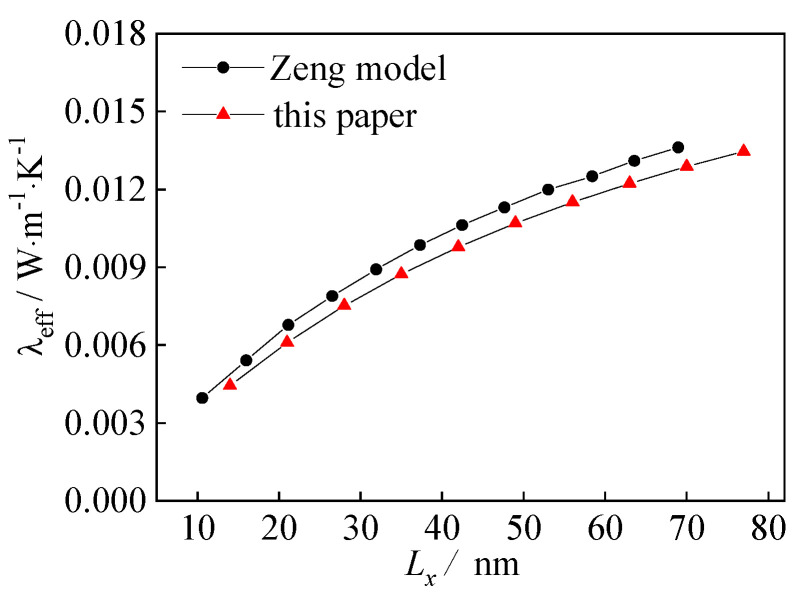
Verification of numerical simulation results.

Set $n=\Delta x_{g}/\Delta x_{s}$ as an integer, that is, the lattice size of the gas phase is several times larger than that of solid phase. Because the lattice size of gas phase and solid phase is different, some lattices will coincide on the virtual boundary, and some lattices will disperse on the virtual boundary, as shown in Figure 5. Because the lattice size of the gas-solid two-phase is different, the time step of the gas-solid two-phase also has a special relationship. If the gas-phase region evolves from time $t_{0}$ to time $t_{g}=t_{0}+\Delta t_{g}$, the solid-phase region needs to evolve *n* time steps: $t_{s}=t_{0}+\Delta t_{s}$, $t_{s}=t_{0}+2\Delta t_{s}$, ⋯, $t_{s}=t_{0}+n\Delta t_{s}$. The physical quantities on lattice *A* at the virtual boundary time *t* are calculated by the second-order interpolation method to ensure the second-order accuracy of the calculation results. Since there is no temperature gradient in the *y* and *z* directions, and the boundary is slip free adiabatic, the specific implementation steps are as follows:

(1) If $A\in \Omega_{g}\cap \Omega_{s}$, as shown in Figure 5a, then
  (14) $$T\left(A\right)=\frac{1}{n+1}\left[T\left(B\right)+T\left(C\right)\right]$$

Here $C\in \Omega_{g}$ and $B\in \Omega_{s}$ are the nearest lattice points in the vertical direction of the virtual boundary.

(2) If $A\notin \Omega_{g}$,$A\in \Omega_{s}$, as shown in Figure 5b, then
  (15) $$T\left(A\right)=\frac{1}{n+1}\left[\alpha T\left(C\right)+\left(1-\alpha \right)T\left(D\right)+nT\left(B\right)\right]$$

Here $D\in \Omega_{g}$, $\alpha =\left|XC\right|/\left|DC\right|$.

The gas-solid two-phase coupling is performed by the second-order interpolation method. According to the region splitting, the gas-solid two-phase sub-regions are calculated by their respective LBM models, and the coupling calculation of the two regions is made by counting the macroscopic physical parameters, *T*, at the interface. Finally, the heat transport process of the whole gas-solid is obtained.

## 4. Program Verification

### 4.1. Gas-Phase Sub-Area Verification

Figure 5 shows the numerical simulation results of the thermal conductivity of the gas-phase region at the pore scale of 10–80 nm, which is compared with the Zeng model [19] under the same conditions.

It can be seen from the figure that the simulation results of this paper are basically consistent with the variation of the theoretical analysis results of the Zeng model, which is slightly smaller than the Zeng model. This is because the potential was weak, and it only considered pairwise interaction between atoms. As a result, the simulation results deviated from this paper in 60~70 nm, although they have the same change trend. Hence, the reliability of the LBM program could be proved.

### 4.2. Solid-Phase Sub-Area Verification

**Figure 6:**
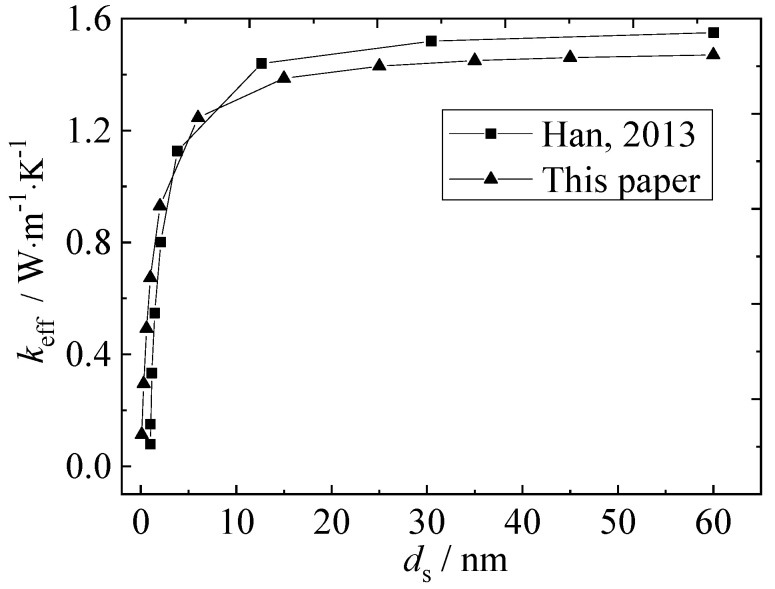
Validation of solid-state numerical simulation.

Figure 6 shows a comparison between the simulation results of thermal conductivity of the solid-phase region and the results of reference [15] under the same conditions. It can be seen from the figure that the simulation results of the solid-phase region in this paper are basically consistent with the variation trend of reference [15].

When the characteristic scale of the solid-phase region is between 0 and 10 nm, the thermal conductivity shows a significant jump, showing the scale-effect characteristics, while the thermal conductivity is close to 1.47 W·m^−1^·K^−1^ of silica in the macroscopic state at 10–60 nm.

## 5. Simulation Results and Analysis

**Figure 7:**
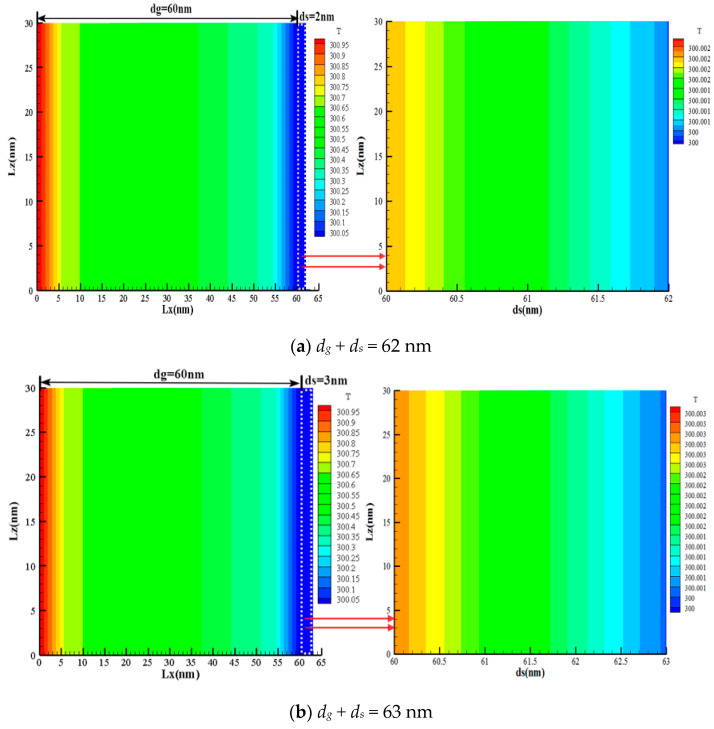

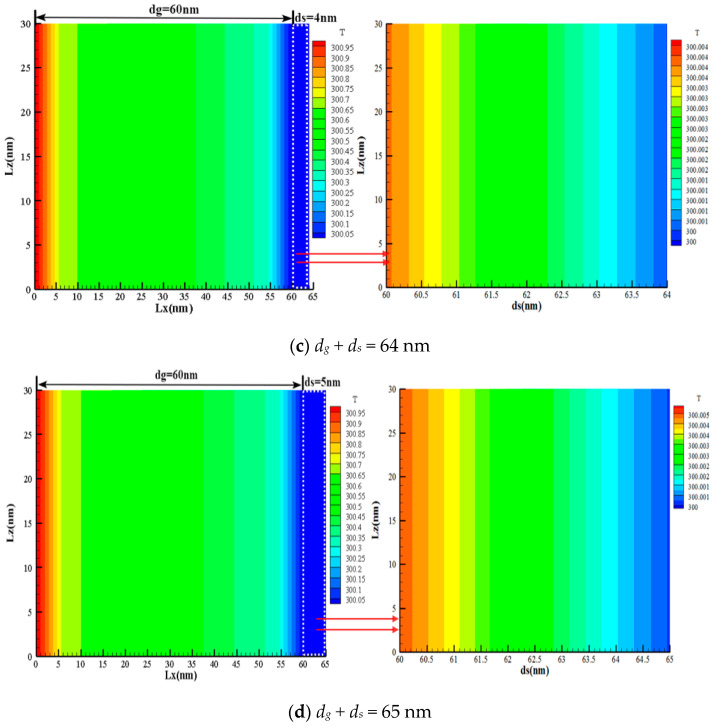
Cloud map of steady temperature distribution of gas-solid coupling.

Figure 7 is a steady-state temperature distribution cloud diagram of a two-dimensional gas-solid center section, in which the gas-phase region *d_g_* is 60 nm, and the solid-phase region *d_s_* is 2 nm, 3 nm, 4 nm, and 5 nm, respectively. Because the solid-phase scale is much smaller than the gas-phase scale, the solid-phase region is presented in the form of enlarged views. From the temperature distribution profiles in Figure 7a–d, since the gas-phase scale is 60 nm, it can be seen that the gas-phase characteristic scale is smaller than the average free path of the gas molecule and the discontinuous temperature diffusion mainly concentrated at the boundary of *x* = 0 nm and *x* = 60 nm, while the temperature distribution in the central region is relatively uniform. From the corresponding temperature distribution diagram of the solid-phase region, it can be seen that the scattering between phonons is dominant because the characteristic scale of the solid phase is much larger than the average free path of the silica phonons at 0.6 nm. At the same time, the characteristics of the concentrated distribution of the solid-phase boundary temperature are weakened. With the increase in the characteristic scale of the solid phase, the transition between the different parts of the temperature distribution becomes more uniform and tends to the temperature diffusion phenomenon under the macroscopic state.

**Figure 8:**
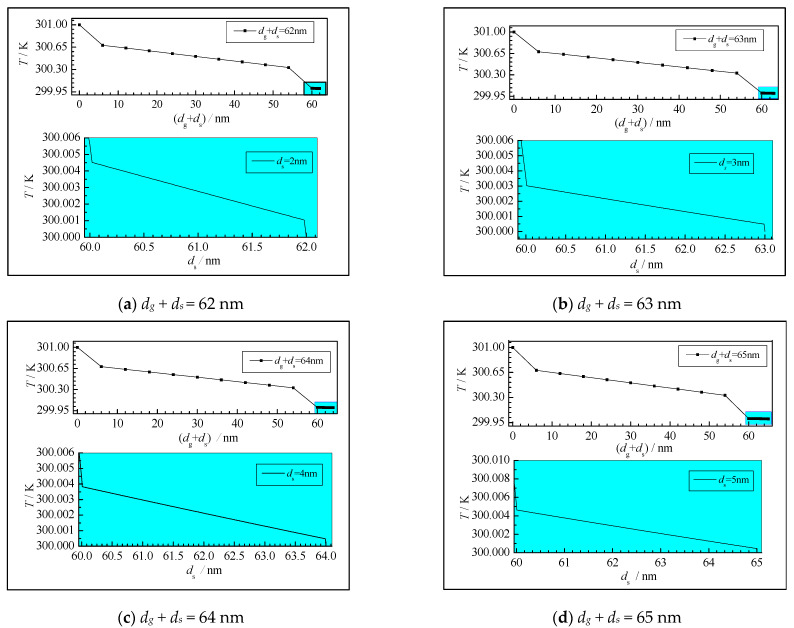
Steady temperature distribution curve of two-dimensional gas-solid center section.

Figure 8 shows the two-dimensional steady-state temperature distribution of the gas-solid central section of the gas-solid coupled heat conduction model. It can be seen from the graph that the gas-phase region *d_g_* is 60 nm, and since the gas-phase characteristic scale is small, the free movement of the gas molecules is severely restricted, which hinders the thermal transport of the heat molecules to the cold molecules, and a strong boundary scattering effect occurs. Therefore, the temperature jump is obvious at the boundary *x* = 0 nm and *x* = 60 nm. It can be seen from the large graph of the respective solid-phase region that there is a significant temperature jump at the interface of gas-solid coupling *x* = 60 nm, and The temperature jump of the gas-phase region is significantly higher than the temperature jump of the solid-phase region. This is because the characteristic scale of the solid-phase region is much larger than the average free path of the silica phonons, at which time the thermal transport between the phonons plays a dominant role, and the scattering between the phonons and the boundaries is relatively weakened. Hence, the temperature jump between the two boundaries is weakening.

**Figure 9:**
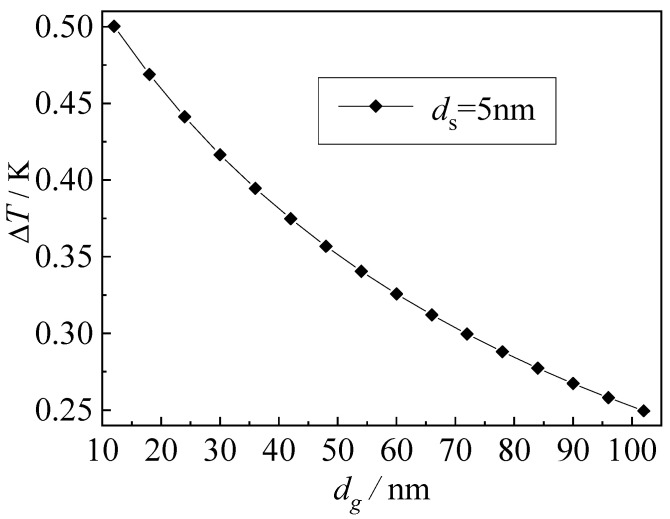
Temperature jump at gas-solid interface.

Figure 9 is a temperature jump diagram at the gas-solid interface when the solid-phase region is 5 nm. It can be seen from the graph that the temperature jump at the interface of the gas-solid coupling decreases with the increase in the gas-phase scale. The temperature jump at 10–70 nm decreases sharply when it reaches 70 nm, and the decrease amplitude begins to be gentle.

This is because when the gas-phase scale is smaller than the mean free path of the gas molecules, the boundary scattering effect is obvious, and the temperature jump at the boundary will be more violent. With the increase in the gas-phase scale, the discontinuous temperature distribution at the boundary caused by the boundary scattering effect will gradually weaken at same time, leading to the decrease in the boundary temperature jump value.

**Figure 10:**
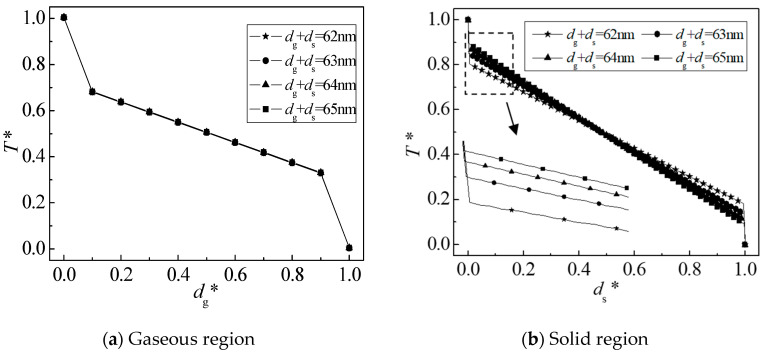
Dimensionless gas-solid temperature distribution. * dimensionless parameters.

Figure 10 is a schematic diagram of the dimensionless temperature distribution of the gas-solid two-phase system. As shown in Figure 10a, since the gas-phase characteristic scale is 60 nm, the temperature distributions in the dimensionless gas-phase regions in the four cases are in good agreement, and the boundary temperature jumps are consistent; as shown in Figure 10b, the temperature jumps in the solid-phase region decrease with the increase in the solid-phase scale, and the temperature diffusion is more and more close to the macroscopic heat transport characteristics. This is because the solid-phase scale is larger than the average free path of phonon 0.6 nm. With the further increase in the solid-phase scale, the scattering effect between the phonon and the boundary will gradually disappear, the scattering between phonon and phonon will be enhanced, and the energy transfer will be smoother. Hence, the macroscopic thermal transport characteristics will gradually appear.

**Figure 11:**
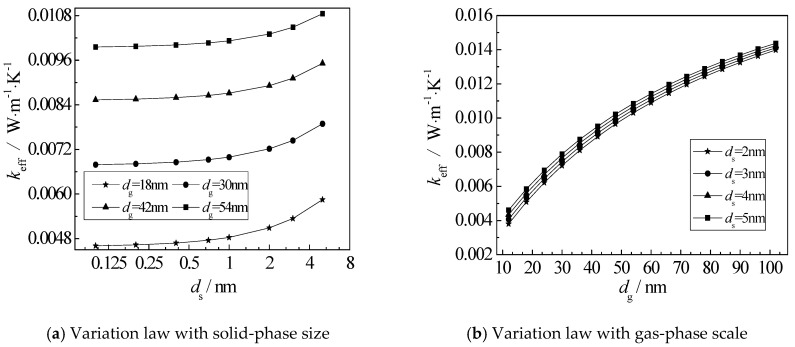
Dependence of Coupled Thermal Conductivity on Solid-Gas Scale.

Figure 11 shows the relationship between the gas-solid coupling thermal conductivity and the solid-gas scale. It can be seen from Figure 11a that when the solid-phase scale is less than 1 nm, the increase in thermal conductivity is small, and when the solid-phase scale is greater than 1 nm, the thermal conductivity begins to increase rapidly. As can be seen from Figure 11b, when the gas-phase scale is between 10 and 70 nm, the increase in thermal conductivity is larger, and when the gas-phase scale reaches about 70 nm, the change trend of thermal conductivity begins to be stable. 

This is because the characteristic scales of the gas-solid two phases are smaller or closer to the mean free path of their molecules and phonons, and boundary scattering will severely restrict the heat transport between the molecules and phonons, showing the obvious scale effect. With the increase in scale, scattering will rapidly weaken and thermal conductivity will correspondingly increase.

## 6. Conclusions

In this paper, the gas-solid coupling heat conduction model of nanoporous aerogels is established. Based on the theory of regional splitting, the gas-solid two-phase simulation is carried out by using the respective LBM. The second-order interpolation is used to transfer temperature at the gas-solid interface. Finally, the internal temperature distribution and the variation of thermal conductivity of gas-solid coupling are systematically studied. The main conclusions are as follows:

(1) When the gas-phase scale is 60 nm, the boundary of the gas-phase region shows obvious discontinuous temperature distribution characteristics, and the temperature jump is obvious; when the transition of the temperature distribution in the solid-phase region is gradually uniform, the solid-phase scale changes from 2 nm to 5 nm.

(2) When the solid-phase scale is 5 nm, the temperature jump at the gas-solid interface decreases with the increase in the gas-phase scale. The temperature jump decreases sharply when the gas-phase scale is 10–70 nm and begins to change gently when the gas-phase scale reaches 70 nm.

(3) The variation of thermal conductivity of gas-solid coupling with solid-phase scale is basically the same. The variation range of thermal conductivity is relatively small when the solid-phase scale is less than 1 nm. When the solid-phase scale is larger than 1 nm, the variation range of thermal conductivity begins to increase rapidly.

(4) The thermal conductivity of gas-solid coupling increases gradually with the increase in the gas-phase scale. The thermal conductivity increases greatly between 10 and 70 nm. When the thermal conductivity reaches about 70 nm, the increase in thermal conductivity begins to slow down gradually.

## Data Availability

The study did not report any data.
